# Supplementary material for: MetPC: Metabolite Pipeline Consisting of Metabolite Identification and Biomarker Discovery Under the Control of Two-Dimensional FDR
Source: Metabolites. 2019 May 25;9(5):103. doi: 10.3390/metabo9050103 (PMC6572057; doi:10.3390/metabo9050103)
Supplement: Supplementary file 1 [file metabolites-09-00103-s001.pdf]

Supplementary metarial: MetPC: Metabolite Pipeline Consisting of metabolite identification and biomarker discovery under the control of two-dimensional FDR

Jaehwi Kim, Jaesik Jeong*

1 Metabolite Identification

Given MS(Mass Spectrum) data, we can easily perform metabolite identification using the MetPC package. To use the MetPC package, three variables ('Name', 'Area', and 'Spectra') should be included in the data. Suppose that we have two data sets: sample and library.

```
sdata <- read.csv("Standard_1.csv", header=T)
ldata <- read.csv("Standard_2.csv", header=T)
sdata <- data.frame(Name=sdata$Name, Area=sdata$Area, Spectra=sdata$Spectra)
ldata <- data.frame(Name=ldata$Name, Area=ldata$Area, Spectra=ldata$Spectra)
```

The following figure shows part of the raw data, which were obtained from two dimensional GCxGC/TOF-MS.

1	Name	CAS	1st Dimen	2nd Dimei	Area	Similarity	Reverse	Probability	UniqueMa	Quant Ma	Purity	Concerns	S/N	Spectra
2	1,3-Butadiene, 1,1,2,3,4,4-	87-68-3	1014.23	1.439	14780	740	777	9585	227	227		NaN	230.69	47:5620 225:3381 83:
3	1,3-Butadiene, 1,1,2,3,4,4-	87-68-3	1019.22	1.353	74021	794	805	9762	225	225		NaN	633.37	47:10642 118:6149 8
4	1,3-Butadiene, 1,1,2,3,4,4-	87-68-3	1059.19	1.432	8178688	847	852	9738	225	225		NaN	52136	47:658651 118:47704
5	1,3-Cyclopentadiene, 1,2,	77-47-4	1254.05	1.518	752296	779	821	9872	239	239		NaN	5935.3	60:92440 95:86048 2
6	1-Propanamine, N-nitros	621-64-7	804.388	1.544	14953951	887	926	9550	70	70		NaN	18492	43:1144582 70:10020
7	2-Cyclohexen-1-one, 3,5,	78-59-1	889.324	1.617	38036173	929	930	9392	82	82		NaN	40623	82:1868875 39:67229
8	Acenaphthene	83-32-9	1483.87	2.099	25548064	953	959	8856	153	153		NaN	122105	153:1913533 154:163
9	Aniline	62-53-3	649.506	1.723	24118755	934	935	5214	93	93		NaN	27485	93:1235149 66:89465
10	Aniline	62-53-3	709.46	1.597	80046	638	810	5814	93	93		NaN	212.22	93:9030 66:4932 65:2
11	Azobenzene	103-33-3	1673.73	2.092	34555052	914	918	9055	77	77		NaN	28165	77:2363922 51:12497
12	Benz[a]anthracene	56-55-3	2608.02	3.313	6624635	921	944	5470	228	228		NaN	36975	228:313393 226:1023
13	Benz[a]anthracene	56-55-3	2613.01	3.373	62768	738	887	5368	113	113		NaN	452.49	228:34595 226:10325
14	Benz[a]anthracene	56-55-3	2618.01	3.412	8739589	917	939	6199	228	228		NaN	31432	228:264422 226:9231
15	Benzenamine, N-phenyl-	122-39-4	1663.73	2.264	12336754	932	940	4920	169	169		NaN	51618	169:665759 168:5101

Figure 1: Part of raw data from two dimensional GCxGC/TOF-MS

Prior to the metabolite identification, peak merging is done by choosing the peak with the largest area.

```
# Peak merging
psdata <- pmerge(sdata)
pldata <- pmerge(ldata)
```

After peak merging, we calculate some statistics, which are inputs for our statistical hierarchical model. That is, the dissimilarity score (S), and the competition scores (b_j and b_j^*) are calculated by using the **cal_ob** function.

```
# Calculate competition/dissimilarity score
obdata <- cal_ob(psdata$Spectra, pldata$Spectra)
```

1.1 Metabolite identification: MetID

We perform identification by calling the **MetID** function, which requires some input such as dissimilarity score, competition score and initial parameters.

```
# Metabolite identification
MetID(500, pldata, 0.8, obdata, muT=2, muF=15, muF2=50, sigmaT=3, sigmaF=15, sigmaF2=50)
```

The first argument 500 is the number of iterations of the EM algorithm and 0.8 is used as the cutoff of the confidence measure. **pldata** is library data after peak merging and **obdata** is score calculated by using sample and library data. The last 6 arguments are initial values of mean and variance in three component normal mixture model. After performing the process of identification, we get the results below:

```
> result
                                    Name Confidence
1                             Octadecane  0.9999996
2                               Eicosane  0.9994737
3                               Undecane  0.9994674
4                                 Decane  0.9994534
5     2-Cyclohexen-1-one, 3,5,5-trimethyl-  0.9994470
6                       Phenol, 3-methyl-  0.9994220
7                             Hexadecane  0.9994173
8                            Heptadecane  0.9994067
9              Phenol, 4-chloro-3-methyl-  0.9994010
10                                Nonane  0.9993744
11                  Benzenamine, N-phenyl-  0.9993550
12                               Aniline  0.9993534
13                  Phenol, 2,4-dichloro-  0.9993491
14                                Phenol  0.9993478
15                     Dimethyl phthalate  0.9993340
16                           Pentadecane  0.9993321
17                           Heneicosane  0.9993309
18                          Dibenzofuran  0.9993276
19                              Dodecane  0.9993159
20                        Benzyl Alcohol  0.9993111
```

Figure 2: Result of metabolite identification

Figure 2 provides the top 20 identification results in terms of confidence measure. All lists are provided in Supplementary Materials II.

1.2 Parameter estimation by the EM-algorithm

For parameter estimation, we assume that score density belongs to the two- or three-component normal mixture. Thus, we implemented three functions: **estpar_tf**, **estpar_ttf**, **estpar_tff**. In case of two-component normal mixture, **estpar_tf** is used. For three-component normal mixture, we consider two different scenarios. That is, two component normal mixture can be considered for true score density or false score density depending on the situation. **estpar_ttf** is used when the distribution of true score is a mixture model while **estpar_tff** is used when the distribution of false score is a mixture model. As a quick check for the density estimation, we suggest to compare it with the kernel density estimator.

For illustration purpose, we here used **estpar_tff** to estimate the parameters and considered 500 iterations of the EM algorithm. The following code generate Figure 3, which provides trace plots for four parameter estimates selected.

```
pars <- estpar_tff(500, obdata, muT=2, muF1=15, muF2=50, sigmaT=3, sigmaF1=15, sigmaF2=50)
plot_pars(pars, 500)
```

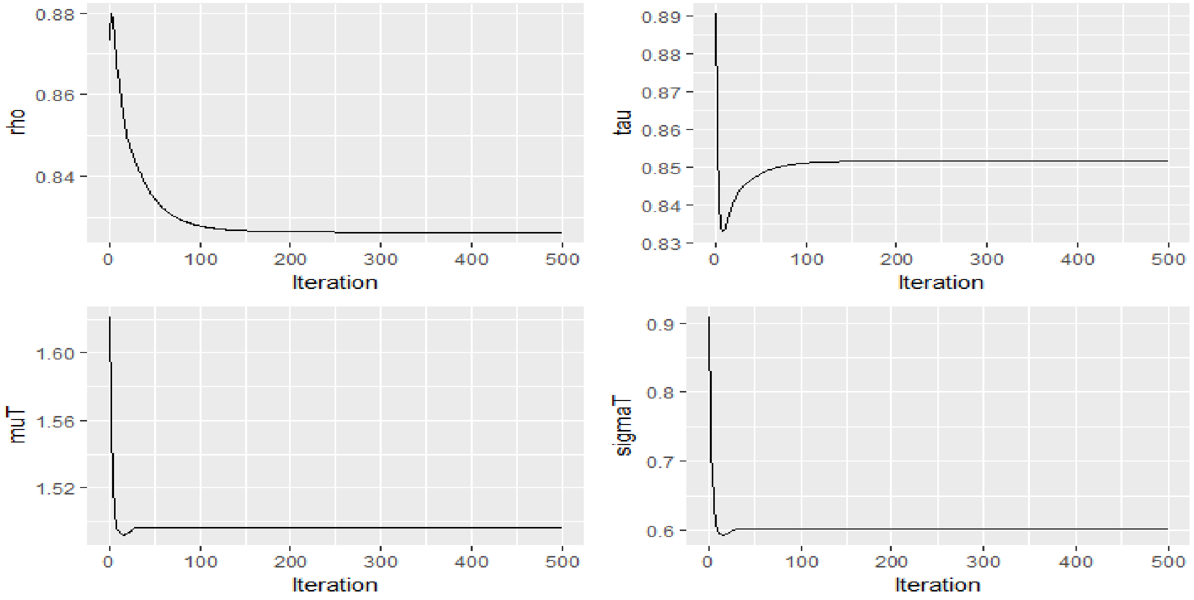

Figure 3: Trace plot of parameter estimates through the EM-algorithm. parameter ρ (top left), parameter τ (top right), parameter μ_T (bottom left), and parameter σ_T^2 (bottom right).

For graphical representation, we used the package **ggplot2**, which can be easily downloaded from the **CRAN**. Based on the plot, some performance measures can be checked, for example, convergence rate of the EM algorithm.

1.3 Kernel density estimator

Kernel density estimator (non-parametric version) is used for two different purposes. It is first used when deciding the type of normal mixture: two- or three-component normal mixture. Also, it can be used to check the accuracy of parameter estimates by looking at the overlap of two density estimates.

```
fT <- pars$pis[500,1]*dnorm(seq(0,90,0.1),pars$muT[500],sqrt(pars$sigmaT[500]))
fF1 <- pars$pis[500,2]*dnorm(seq(0,90,0.1),pars$muF1[500],sqrt(pars$sigmaF1[500]))
fF2 <- (1-pars$pis[500,1]-pars$pis[500,2])*dnorm(seq(0,90,0.1),pars$muF2[500],sqrt(pars$sigmaF2[500]))
kd <- data.frame(Score=obdata$S[,3])
pd <- data.frame(score=seq(0,90,0.1),density=fT+fF1+fF2)

ggplot(kd,aes(x=Score))+geom_density(aes(color="Kernal density estimator"),size=1) +
  geom_line(data=pd,aes(x=score,y=density,color="Parametric density estimator"),size=1,linetype="dashed") +
  scale_colour_manual(NULL,values = c("black", "violetred")) +
  theme(legend.background = element_rect(size=2, linetype="solid", colour ="darkred"),
    legend.position = c(0.6,0.6), legend.text=element_text(size=30),
    axis.text.x = element_text(size=20), axis.text.y = element_text(size=20),
    axis.title.x = element_text(size=20), axis.title.y = element_text(,size=20))
```

Two types of density estimates are included in Figure 4. It seems that normal mixture density estimate overlaps the kernel density estimate very well.

2 Biomarker Discovery

The discovery of biomarker metabolites is done under the control of two dimensional local false discovery rate (2d-fdr), which was implemented by Ploner et al. (2006). For biomarker discovery, another data set is considered. The data is pre-processed before it is used, i.e., log-transformation and standardization.

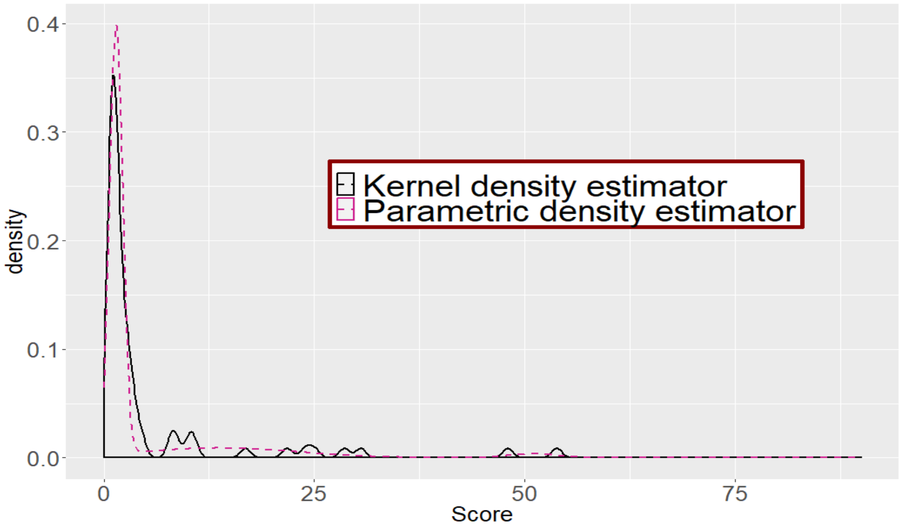

Figure 4: Non-parametric vs Parametric density estimate. Black line is kernel density estimate and red line is parametric density estimation.

```
dat <- read.table("omija.txt", header=F)
colnames(dat) <- rep(c("chinese","korean"), c(27,30))
tdat <- dat[21:(nrow(dat)-20),] # removing top and bottom
ctdat <- log(1+tdat)
for(i in 1:57){ ctdat[,i]=ctdat[,i]/sum(ctdat[,i]) }
```

The following code shows how to conduct biomarker discovery by using the **fdr2d** function.

```
# 2d-fdr
fdr <- fdr2d(ctdat, colnames(ctdat), nperm=500)
summary(fdr)
```

nperm is the number of permutations of group labels that is used for the estimation of 2d-fdr. Here, we considered 500 permutations. The following code shows how to generate two plots: tornado and volcano plot.

```
Tornadoplot(fdr, main="Tornado plot", label=T, cex=2, cex.main=2, col="gray50", lcol="darkred")
Volcanoplot(fdr,55, main="Volcano plot", label=T, cex=2, cex.main=2, col="gray50", lcol="darkred")
```

In the code above, we used 55 as the degree of freedom, which is used to set the color of the contour line. Figure 5 provides two plots: tornado and volcano plot. The only difference is y-axis: log(se) v.s. -log(p-value).

3 Software availability

The current version of bioinformatics tool is available at https://github.com/jjs3098/CNU-Bioinformatics-Lab. Furthermore, example data used in our paper are provided as well. The snapshot of the website is given in Figure 6.

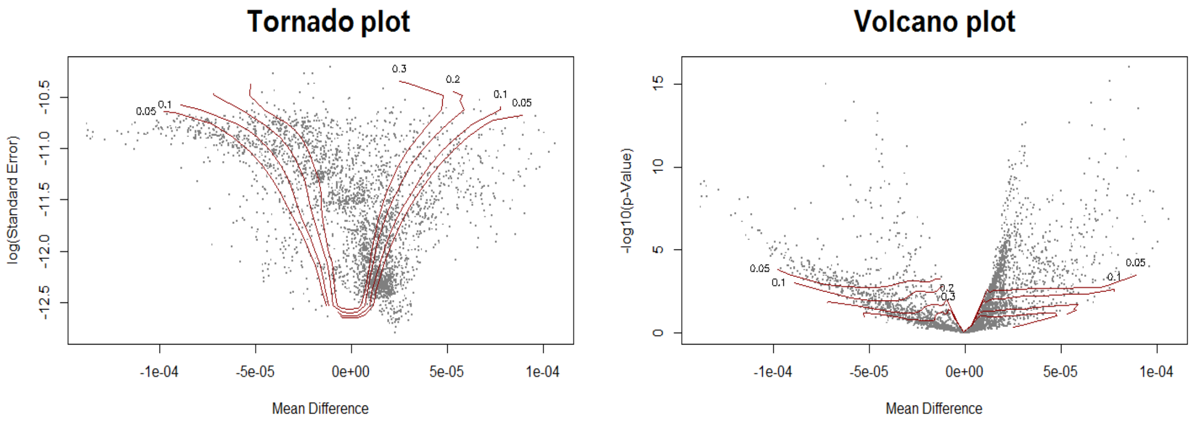

Figure 5: The tornado plot (left) and the volcano plot (right)

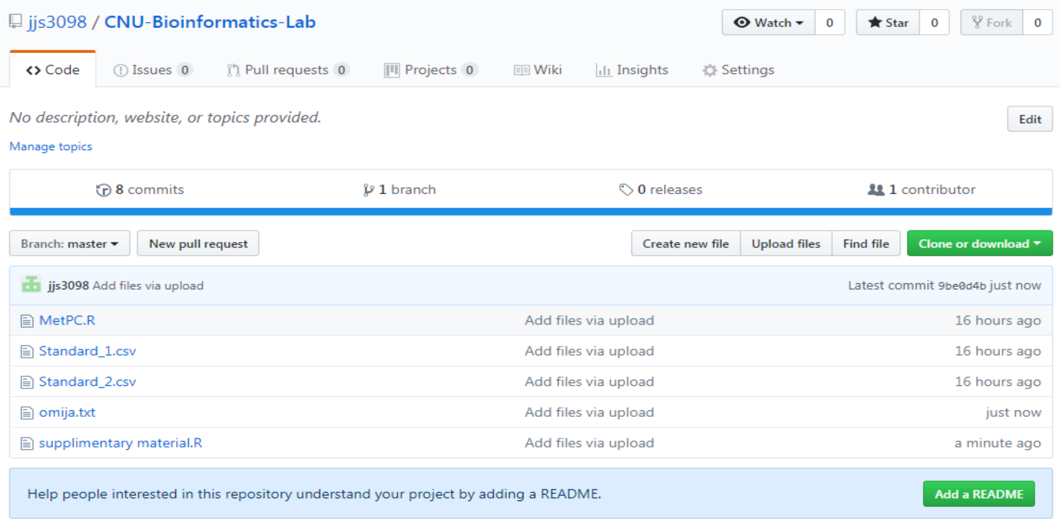

Figure 6: Snapshot of github website
